# Histone demethylase KDM4B accelerates the progression of glioblastoma via the epigenetic regulation of MYC stability

**DOI:** 10.1186/s13148-023-01608-4

**Published:** 2023-12-13

**Authors:** Zhongze Wang, Huarui Cai, Zekun Li, Wei Sun, Erhu Zhao, Hongjuan Cui

**Affiliations:** 1 State Key Laboratory of Resource Insects, Medical Research Institute, Southwest University, No.2 Tiansheng Road, Beibei district, Chongqing, 400715 China; 2 State Key Laboratory for Cellular Stress Biology, School of Life Sciences, Faculty of Medicine and Life Sciences, Xiamen University, Fujian, 361102 China; 3 Jinfeng Laboratory, Chongqing, 401329 China; 4 Chongqing Engineering and Technology Research Center for Silk Biomaterials and Regenerative Medicine, Chongqing, 400716 China

**Keywords:** KDM4B, MYC, miR-181d-5p, Epigenetic regulation, Tumor progression

## Abstract

**Background:**

Glioblastoma (GBM) is the most malignant and invasive human brain tumor. Histone demethylase 4B (KDM4B) is abnormally expressed in GBM, but the molecular mechanisms by which KDM4B affects the malignant tumor progression are not well defined.

**Methods:**

GBM cell lines and xenograft tumor samples were subjected to quantitative PCR (qPCR), Western blot, immunohistochemical staining (IHC), as well as ubiquitination, immunoprecipitation (IP), and chromatin immunoprecipitation (ChIP) assays to investigate the role of KDM4B in the progression of GBM.

**Results:**

Here, we report that KDM4B is an epigenetic activator of GBM progression. Abnormal expression of *KDM4B* is correlated with a poor prognosis in GBM patients. In GBM cell lines, KDM4B silencing significantly inhibited cell survival, proliferation, migration, and invasion, indicating that KDM4B is essential for the anchorage-independent growth and tumorigenic activity of GBM cells. Mechanistically, KDM4B silencing led to downregulation of the oncoprotein MYC and suppressed the expression of cell cycle proteins and epithelial-to-mesenchymal transition (EMT)-related proteins. Furthermore, we found that KDM4B regulates MYC stability through the E3 ligase complex SCF^FBXL3+CRY2^ and epigenetically activates the transcription of CCNB1 by removing the repressive chromatin mark histone H3 lysine 9 trimethylation (H3K9me3). Finally, we provide evidence that KDM4B epigenetically activates the transcription of miR-181d-5p, which enhances MYC stability.

**Conclusions:**

Our study has uncovered a KDM4B-dependent epigenetic mechanism in the control of tumor progression, providing a rationale for utilizing KDM4B as a promising therapeutic target for the treatment of MYC-amplified GBM.

**Supplementary Information:**

The online version contains supplementary material available at 10.1186/s13148-023-01608-4.

## Introduction

Glioblastoma (GBM) is classified as grade 4 glioma by the WHO and is considered the most malignant and invasive human brain tumor [1]; approximately 90% of GBMs are primary tumors, while the remaining tumors represent secondary GBM [2]. The prognosis of patients with GBM is extremely poor. The 5-year survival rate after diagnosis is approximately 6.8%, which is much lower than the 35.8% five-year survival rate of patients with other malignant brain tumors [3]. Currently, the most effective clinical treatment approach is tumor resection combined with radiotherapy and adjuvant temozolomide treatment, but the prognosis remains poor [4]. GBM is almost impossible to successfully treat because of its aggressive nature and the difficulty of delivering drugs to the tumor site [5]. Therefore, in recent years, an increasing number of researchers have focused on the development and application of targeted drugs, and are now eager to efficiently translate these findings to the clinic [6]. With our research, we aimed to screen and identify effective biomarkers, clarify the pathogenesis of GBM, and explore suitable clinical therapeutic targets for GBM.

Histone demethylase 4B (KDM4B), which is usually localized in the nucleus, regulates the transcriptional activity of target genes by removing methyl groups from lysine residues in histone tails [7]. The Histone demethylase 4 (KDM4) family consists of six members, KDM4A-KDM4F, among which KDM4A-KDM4D are highly expressed and play a central regulatory role in a variety of cancers, including prostate cancer, breast cancer, and neuroblastoma. Therefore, KDM4A-KDM4D have gradually been considered potential broad-spectrum therapeutic targets [8]. In cancer, abnormal expression of KDM4B leads to excessive opening of chromatin and promotes oncogene transcription [9]. Moreover, KDM4B can interact with oncogenes to participate in downstream signaling pathways and play an intermediate regulatory role in prostate cancer, breast cancer, and neuroblastoma [10–13]. Currently, the expression of KDM4B in GBM and the specific regulatory mechanism have not been reported..

The myelocytomatosis (MYC) protein is a transcription factor belonging to the MYC oncoprotein family. The MYC protein mainly contains two key domains. One is the Box domain at the N-terminus, which is generally able to bind various effector proteins, including TRRAP and P400, which mediate chromatin remodeling and modification [14, 15]. In neuroblastoma, the histone demethylase KDM4B was also found to bind to the acidic central region of the N-MYC protein, thereby coregulating the downstream MYC signaling pathway [11]. The other domain is the basic helix-loop-helix-leucine-zipper (BHLH-LZ) domain at the C-terminus, which can form a "zipper" structure with the chaperone protein MAX, and the resulting complex binds to DNA recognition sequences to exert transcriptional regulation effects [16]. Existing studies have shown that MYC mainly plays a role in transcriptional regulation and provides a platform for interactions with other cofactors, leading to its pleiotropic effect, which plays a role in promoting proliferation and metabolic reprogramming and enhancing chemotherapeutic resistance in GBM [17]. Therefore, strict restriction of MYC expression in GBM may have great therapeutic value.

Furthermore, studies have revealed that ubiquitin-mediated protein degradation is an effective mechanism by which GBM cells regulate the expression of the MYC protein and inhibit the function of MYC [18] Currently known E3 ubiquitin ligases that directly target MYC include F-box and WD repeat domain containing 7 (FBXW7) [18], F-box and leucine-rich repeat protein 3 (FBXL3) [19], and the carboxyl terminus of HSC70-interacting protein (CHIP) [20]. FBXL3 must interact with cryptochrome circadian regulator 2 (CRY2) to ubiquitinate MYC for degradation [19]. Interestingly, the mRNA levels of these E3 ligases are directly regulated by miR-181d-5p [21]. These E3 ubiquitin ligases act as tumor suppressor genes and are downregulated in tumors to stabilize MYC protein expression and promote tumor development [18, 20, 21]. Our study has identified a novel regulatory relationship between KDM4B and MYC, which provide a new appreciation of KDM4B as an indirect MYC-related therapeutic target in GBM.

## Materials and methods

### Cell lines

GBM cell lines (U87-MG, LN229, A172, U251-MG, and U118-MG) and the normal glial cell line SVGP12 were purchased from American Type Culture Collection. 293FT cells were purchased from Invitrogen. All cells were cultured by a previously described method [18].

### Reagents, plasmids, and microRNA

Lipofectamine 3000 was obtained from Thermo (MA, America). Polybrene was obtained from Yeasen (Shanghai, China). Puromycin and MG132 were obtained from Beyotime (Shanghai, China). Immunohistochemical kits were obtained from ZSGB-Bio (Beijing, China). The microRNA extraction kit was purchased from RIBOBIO (Guangzhou, China). KDM4B (A301-478A) antibodies from Bethyl, Ki67 (9449) antibodies from CST, and MYC (ab32072) antibodies from Abcam were used for immunohistochemistry (IHC). Cycloheximide (CHX) was obtained from Cell Signaling Technology (CST).

The shRNAs targeting KDM4B (shKDM4B#1, shKDM4B#2) were cloned and inserted into the pLKO.1 lentiviral vector. Recombinant plasmids containing human Flag-KDM4B, Flag-KDM4BΔJmjC (G494C, G545C), and HA-Ub were acquired from Youbao Company (Changsha, China) and inserted into pCDH-CMV-MCS-EF1-GFP-Puro. MiR-181d-5p was acquired from RiboBio (Guangzhou, China). All primers for the shRNA sequences are listed in Table 1.

**Table 1:** Primers of shRNA

shKDM4B#1-forward (5′ − 3′)	CCGGCCGGCCACATTACCCTCCAAACTCGAGTTTGGAGGGTAATGTGGCCGGTTTTTG
shKDM4B#1-reverse (5′ − 3′)	AATTCAAAAACCGGCCACATTACCCTCCAAACTCGAGTTTGGAGGGTAATGTGGCCGG
shKDM4B#2-forward (5′ − 3′)	CCGGGCCCATCATCCTGAAGAAGTACTCGAGTACTTCTTCAGGATGATGGGCTTTTTG
shKDM4B#2-reverse (5′ − 3′)	AATTCAAAAAGCCCATCATCCTGAAGAAGTACTCGAGTACTTCTTCAGGATGATGGGC

### Transfection and infection

The experimental procedures were conducted following the methodology outlined in our previous publications [22, 23]. In brief, pLP1, pLP2, and pLP/VSVG were used as packaging plasmids and co-transfected with overexpression plasmids or interference plasmids into 293FT cells with Lipofectamine 3000. After 48 h, the supernatant was collected, and a membrane with a 0.22 μm pore size was used to filter lentivirus particles. GBM cells were seeded in small 60 mm dishes at 33% confluence. After 12 h, lentivirus and normal medium were added to the dishes for infection of GBM cells for 24 h in the presence of polybrene. After 2 infection steps, the cells were placed in a plate and suspended for screening with puromycin. GBM cell lines with stable gene overexpression or knockdown were gradually established.

### Quantitative PCR (qPCR)

Total RNA was extracted with TRIzol reagent (Invitrogen, Carlsbad, CA, USA) for reverse transcription into cDNA using a reverse transcription kit (Yeasen, Shanghai, China). The qPCR was used to measure the transcription level of the genes. Glyceraldehyde-3-phosphate dehydrogenase (GAPDH) was used as an internal reference control for quantitative analysis. The fluorescent dye used for quantification was SYBR Green. MicroRNAs were reverse-transcribed by a microRNA reverse transcription kit (RiboBio, Guangzhou, China). The expression levels of microRNA were then analyzed using a microRNA assay (RiboBio, Guangzhou, China). The microRNA U6 was used as an endogenous control. The primers used for qPCR analysis are listed in Table 2.

**Table 2:** The qPCR primers

KDM4B-forward (5′ − 3′)	AGACGTATGATGACATCGACGA
KDM4B-reverse (5′ − 3′)	CGTAGATCGGGGAGACAAAGG
MYC-forward (5′ − 3′)	GTCAAGAGGCGAACACACAAC
MYC-reverse (5′ − 3′)	TTGGACGGACAGGATGTATGC
CCNB1-forward (5′ − 3′)	TTGGGGACATTGGTAACAAAGTC
CCNB1-reverse (5′ − 3′)	ATAGGCTCAGGCGAAAGTTTTT
FBXL3-forward (5′ − 3′)	AGTGACAACGTC GAGCACAG
FBXL3-reverse (5′ − 3′)	CGGTCGCTACCATTACCAGT
CRY2-forward (5′ − 3′)	GGTGTGGAAGTAGTGACGGAG
CRY2-reverse (5′ − 3′)	GTAGGTCTCGTCGTGGTTCTC
GAPDH-forward (5′ − 3′)	GGAGCGAGATCCCTCCAAAAT
GAPDH-reverse (5′ − 3′)	GGCTGTTGTCATACTTCTCATGG
miR-181d-5p-forward (5′ − 3′)	CCGCTCGAGAACTTGCCAAGGGTTTGGGGGAACA
miR-181d-5p-reverse (5′ − 3′)	CCGGAATTCATGTTCATCTACCAGTTTGCCCACT
U6-forward (5′ − 3′)	TCGCTTCGGCAGCACATA
U6-reverse (5′ − 3′)	TTTGCGTGTCATCCTTGC

### Western blot analysis

Western blots were used to measure protein expression levels. The protein extraction and detection procedures were performed in accordance with our previous article [24]. The following antibodies were used in this experiment: antibodies against KDM4B (8639S), cyclin B1(CCNB1) (4135S), and Flag (14793S) were purchased from CST. Antibodies against MYC (10,828–1-AP), p27 (25,614–1-AP), cyclin dependent kinase 1 (CDK1) (19,532–1-AP), α-Tubulin (66,031–1-Ig), H3K9me3 (39,285), H3 (17,168–1-AP), GAPDH (60,004–1-Ig), HA-Tag (66,006–2-Ig), Snail (130,991–1-AP), Slug (12,129–1-AP), E-cadherin (20,874–1-AP), and matrix metallopeptidase 2 (MMP2) (10,373–2-AP) were purchased from Proteintech. Antibodies against FBXL3 and CRY2 were purchased from Bioss.

### Cell viability assay

Cell viability was measured by the MTT (Sigma-Aldrich, St. Louis, MO, USA) method. Approximately 2000 cells were cultured in each well of 96-well plates and evaluated at 1, 3, 5, and 7 days. For detection, MTT was added, and the plates were incubated in an incubator for 2 h. Then, all the liquid was aspirated, and 200 μl of dimethyl sulfoxide (DMSO) (Sangon Biotech, Shanghai, China) was added for 30 min to dissolve the substrate. A microplate reader (Thermo Fisher, Waltham, MA, USA) was used to measure the absorbance of the cells at 490 nm.

### Plate colony formation experiment

First, 2000 cells per well were cultured in 24-well plates for 7 days. Then, the cells were dyed for 20 min with crystal violet dye (Beyotime, Shanghai, China). Images were scanned with a scanner, and colonies were counted.

### BrdU incorporation assay

First, 10,000 cells were seeded in 24-well plates and cultured for 24 h. Then, 10 g/ml BrdU (Sigma-Aldrich, St. Louis, MO, USA) was added and incubated for 2 h. Then, the cells were fixed with 4% paraformaldehyde for 20 min, treated with 2 M HCl for 30 min to denature the DNA, treated with 1% Triton X-100 for 30 min, and blocked with 5% goat serum (diluted with 0.3% Triton X-100). Next, the primary antibody (Abcam, Cambridge, MA, USA) was added and incubated for 10 h at 4 °C, and the secondary antibody (Abcam, Cambridge, MA, USA) was added and incubated for 2 h. Finally, the cells were incubated with DAPI (Beyotime, Shanghai, China) for 30 min to stain DNA. Images were acquired under a fluorescence microscope, and the positive rate of BrdU incorporation was calculated.

### Flow cytometry

The cell cycle was analyzed by flow cytometry. First, cells were cultured in medium containing 1% serum for 12 h; the medium was then replaced with normal medium for 24 h, and the cells were collected. The cells were fixed with 75% ethanol at 4 °C for 2 days. Next, the cells were washed three times with PBS and then incubated with propidium iodide (PI; BD, San Jose, CA, USA) and RNase A (Sigma-Aldrich, St Louis, MO, USA) at 37 °C for 1 h. All samples were analyzed using a FACS C6 instrument (BD, San Jose, CA, USA) with Cell Quest software.

### Soft agar assay

A soft agar assay was used to evaluate GBM cell tumorigenicity in vitro. The experimental procedures were conducted following the methodology outlined in our previous publications [22, 25].

### Tumor xenografts

Immunodeficient (NOD/SCID) mice approximately 28 days old were purchased from Beijing Weitong Lihua Laboratory Animal Technology Co., Ltd. (Beijing, China). Cells were collected, counted, and resuspended in PBS. Tumor cells were subcutaneously injected into the hind limbs of mice. The tumor volume was measured every 4 days. All animal experiments were performed according to protocols approved by the Institutional Animal Care and Use Committee of Southwest University (IACUC-20220328–02).

### Immunohistochemical staining

First, the tumor mass was dehydrated using a dehydrator (Leica, Germany). Then, the tumor was embedded in paraffin, cut into 5 mm thick slices, spread, and placed on immunohistochemistry slides (ZSGB-BIO). Then, the tumor tissues were deparaffinized and rehydrated, and 0.5% Triton X-100 was added for another hour. The tumor tissues were subjected to antigen repair and treatment with blocking buffer. Antibodies specific for KDM4B, Ki67, MYC, FBXL3, and CRY2 were diluted in phosphate-buffered saline (PBS), added to the tumor tissue sections, and incubated at 4 °C. After 8 h, color was developed by using secondary antibodies. Finally, the stained tumor tissue was dehydrated and sealed for imaging.

### Ubiquitination assay

The shKDM4B and ubiquitin (HA-Ub) plasmids were transfected into GBM cells with Lipofectamine 3000. Two days later, MG132 was added for 7 h. Finally, the cells were collected for subsequent immunoprecipitation (IP) and Western blot analysis.

### Immunoprecipitation

A total of 6 × 10^6^ cells were collected in a tube and 400 μl of IP lysis buffer (Beyotime, Shanghai, China) was added for 2 h on ice. Then, 100 μl of the lysate was used as input. Next, 30 µl of protein A/G magnetic beads (Beyotime) was added to remove contaminants. Then, after centrifugation, the supernatant was transferred to a new tube, and the primary antibody was added for incubation overnight at 4 °C. Then, 40 µl of protein A/G magnetic beads was added. The beads were washed three times with PBS. After the bound protein complexes and input lysate were added to loading buffer, they were incubated at 98℃ for 15 min. Finally, Western blot analysis was performed on the samples.

### Chromatin immunoprecipitation

Chromatin immunoprecipitation (ChIP) was performed with a ChIP assay kit (Beyotime) according to the manufacturer’s instructions. GBM cells were treated with 1% formaldehyde for 10 min, after which the cross-linking was terminated with glycine. The medium was removed, and proteins were then scraped off with PBS containing PMSF. Lysis was performed on ice for 10 min using SDS lysis buffer. Then, the chromosome fragments were sonicated to obtain DNA fragments of approximately 200–1000 bp. The supernatant was removed by centrifugation and ChIP dilution buffer was added. The mixture was incubated with protein A + G agarose/salmon sperm DNA to minimize background noise. The supernatant was equally divided into two tubes: one tube received anti-KDM4B (CST) antibody, while the other tube received IgG as a control. The tubes were incubated overnight at 4° C. Then, protein A + G agarose/salmon sperm DNA were added to each tube and incubated for 1 h at 4 °C. The resulting precipitates were retained by centrifugation and washed sequentially with Low Salt Immune Complex Wash Buffer, High Salt Immune Complex Wash Buffer, and LiCl Immune Complex Wash Buffer. The supernatant was obtained by washing with an appropriate amount of Elution buffer. Finally, qPCR was performed on the purified DNA samples. The relevant primer sequences are presented in Table 3.

**Table 3:** ChIP experimental primers

CCNB1-1536/-1116-F	GGGCGCCTGCCGTCC
CCNB1-1536/-1116-R	TCTTCTGTCATCATAGTTCACCCTC
CCNB1-1153/-789-F	GGCATTCGAGTAGGAGGGTG
CCNB1-1153/-789-R	TGCCCTTTACTCAGATTCAACTATC
CCNB1-794/-293-F	AGGGCATAGAAATATTCCTTACAAG
CCNB1-794/-293-R	AAGGTTAGCCGGGCAAGAGT
CCNB1-310/-1-F	TCTTGCCCGGCTAACCTTT
CCNB1-310/-1-R	GTTCCGCCGCAGCACG

### Statistical analysis

All data are presented as mean ± standard deviation (SD) and were analyzed using GraphPad Prism software 6.0 (GraphPad Prism Software Inc., San Diego, CA, USA). Comparisons between the two groups were conducted using Student’s t-test. For multiple comparisons, we employed one-way analysis of variance (ANOVA). Any p value < 0.05 was considered statistically significant.

## Results

### Abnormal expression of KDM4B is an indicator of a poor prognosis in glioma patients

**Fig. 1:**
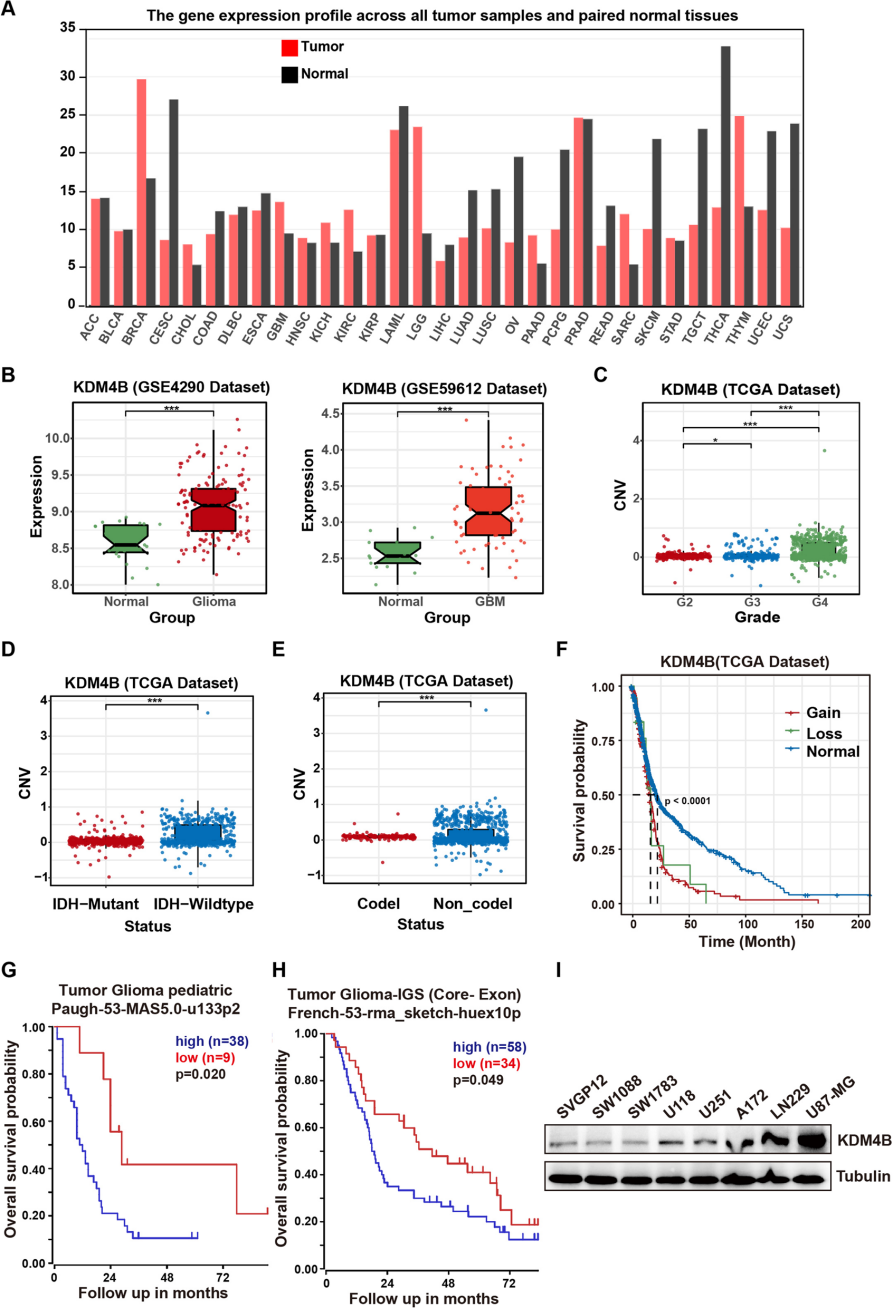
Abnormal expression of KDM4B is an indicator of a poor prognosis in glioma patients. **A** The expression of KDM4B in GBM and many other tumor types was determined using the GEPIA (http://gepia.cancer-pku.cn/index.html) databases. **B** Expression of KDM4B in normal human brain cells versus gliomas and GBM using the Brainbase (https://ngdc.cncb.ac.cn/brainbase/) date. **C** The CNV levels of KDM4B were detected in different grades of gliomas. G2 = Grade II Glioma, G3 = Grade III Glioma, G4 = GBM, CNV = Copy Number Variation. **D** CNV levels of KDM4B were detected between IDH-mutant and IDH-wild-type statuses. **E** CNV levels of KDM4B were detected between the 1p/19q codel and 1p/19q non-codel statuses. **F** Comparison of survival probability in glioma patients among normal, gain and loss of DNA in the CNV regions of KDM4B. **G, H** High KDM4B expression is associated with reduced overall survival in glioma patients. Patient data analyses were conducted online (R2 Genomics Analysis and Visualization Platform), and the resulting figures and log-rank test p values were downloaded. **I** The expression levels of KDM4B in various cell lines including the glial cell line (SVGP12), as well as the LGG cell lines (SW1088, SW1783), and the GBM cell lines (U118, U251, A172, LN229, and U87-MG). *p < 0.05; ***p < 0.001

KDM4B has been found to function as an oncoprotein in a variety of cancers, but such reports in GBM are rare [8]. To preliminarily determine the key role of KDM4B in GBM, we first analyzed the expression and gene level of KDM4B through the GEPIA and BrainBase databases. In the GEPIA database, KDM4B expression levels in both GBM and low-grade gliomas (LGGs) were higher than those in normal tissues (Fig. 1A). In addition, KDM4B expression levels in gliomas and GBM tissues were significantly higher than those in normal tissues in the BrainBase database (Fig. 1B). Genomic aberrations are important independent predictors of disease progression and survival, and these aberrations include single nucleotide variants, translocations, inversions, and copy number variants (CNVs, gain, or loss of DNA) [26]. The CNV level of KDM4B increased with increasing glioma grade and was the highest in GBM (Fig. 1C). IDH wild-type, 1p/19q non-codeletion and MGMT promoter hypomethylation have been shown to be mainly associated with a poor prognosis in GBM patients [27–29]. In patients with these indicators, the CNV level of KDM4B is significantly increased, suggesting that abnormal expression of KDM4B may be related to tumor development (Fig. 1D, E, Additional file 1: Fig. S1). Furthermore, we analyzed the survival probability among patients with no alteration or gain/loss of DNA in the CNV regions of KDM4B. We observed that the control group, which had no loss or gain of DNA copies (considered as the normal group), showed a significantly increased survival probability in glioma patients (Fig. 1 F). Next, we analyzed the expression of KDM4B in glioma patients using the R2 database, and further evidence indicated that abnormal expression of KDM4B is associated with a poor prognosis in glioma patients (Fig. 1G-H). KDM4B expression was detected across 8 cell lines. The U87-MG and LN229 cell lines had a relatively high level of KDM4B expression (Fig. 1I). Therefore, we selected the U87-MG and LN229 cell lines to carry out follow-up experiments. Collectively, these findings suggest that abnormal expression of KDM4B is an indicator of a poor prognosis in glioma patients.

### KDM4B is essential for supporting cancer cell survival, proliferation, migration, and invasion

**Fig. 2:**
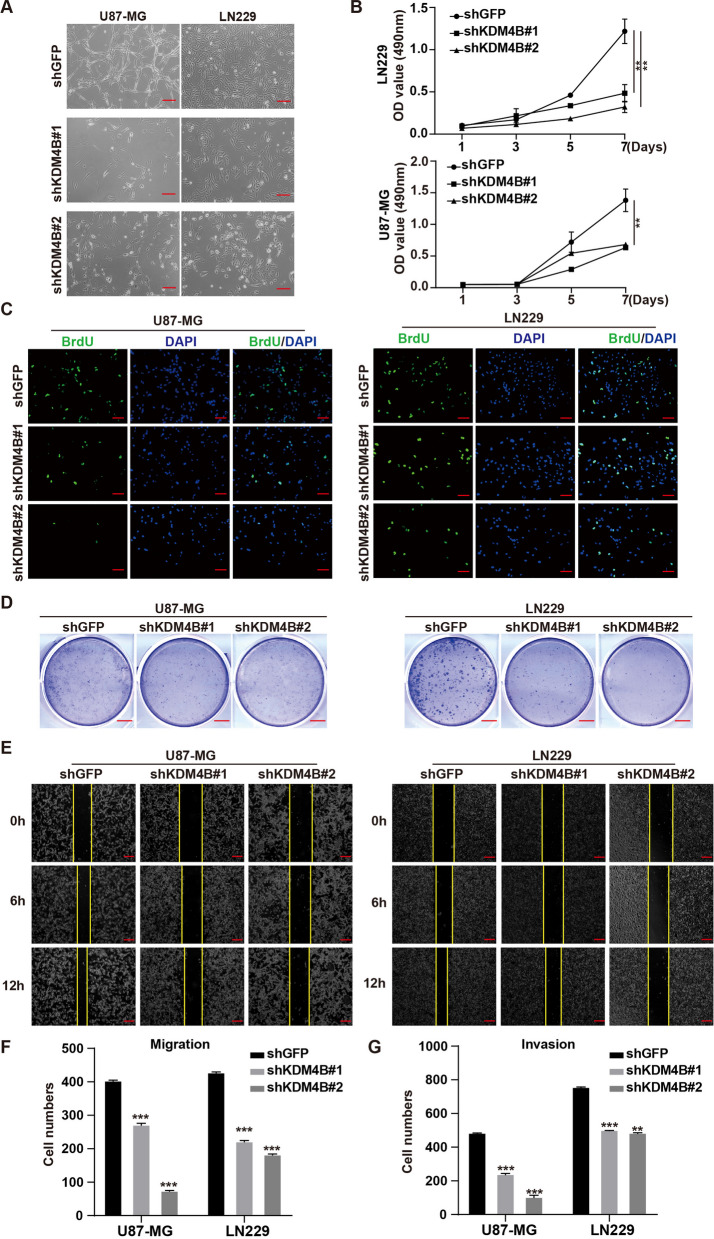
KDM4B is essential for supporting cell survival, proliferation, migration, and invasion. **A** Morphology of cells was observed after knocking down KDM4B. **B** MTT curves of GBM cells after KDM4B knockdown. **C** Cells positive for BrdU staining after KDM4B knockdown are shown. **D** Analysis of colony formation in GBM cells after KDM4B knockdown. **E** The migration ability of KDM4B-knockdown GBM cells was measured by the wound healing test. **F-G** The migration and invasion ability of KDM4B-knockdown GBM cells were detected by transwell assays. **p < 0.01; ***p < 0.001; ns, not significant

To verify the effect of KDM4B on GBM cell proliferation, we successfully knocked down KDM4B in U87-MG and LN229 cells (Additional file 1: Fig. S2A-B). We observed a significant decrease in cell survival after KDM4B knockdown (Fig. 2A and Additional file 1: Fig. S2C). Next, we detected the effect of KDM4B on GBM cell proliferation. The MTT assay results showed that KDM4B knockdown significantly inhibited GBM cell proliferation (Fig. 2B). The number of cells with active DNA replication was significantly reduced, as shown by the BrdU incorporation assay (Fig. 2C and Additional file 1: Fig. S2D). Subsequently, a plate colony formation assay was used to assessed single-cell colony formation ability; the results showed that colony formation was suppressed (Fig. 2D and S2E). These data suggested that KDM4B is essential for cell proliferation in GBM. Invasiveness, which is essential for metastasis, is an important characteristic feature of GBM cells; although distant metastases are extremely rare, GBM causes intracranial metastases that form aggressive secondary lesions, leading to a poor prognosis [30]. To determine whether KDM4B is essential for GBM cell migration and invasion, wound healing assays and transwell assays with or without Matrigel were performed. After KDM4B knockdown, the migratory ability of GBM cells was significantly reduced, as indicated by the wound healing assay (Fig. 2E and Additional file 1: Fig. S2F). Similarly, shKDM4B cells showed a weaker ability to pass through the porous membrane or both the membrane and the Matrigel, suggesting that KDM4B knockdown significantly inhibited cell migration and invasion (Fig. 2F-G). Collectively, these results reveal that KDM4B is essential for supporting cell migration and invasion in GBM.

### KDM4B is required for anchorage-independent cell growth and tumorigenesis in GBM

**Fig. 3:**
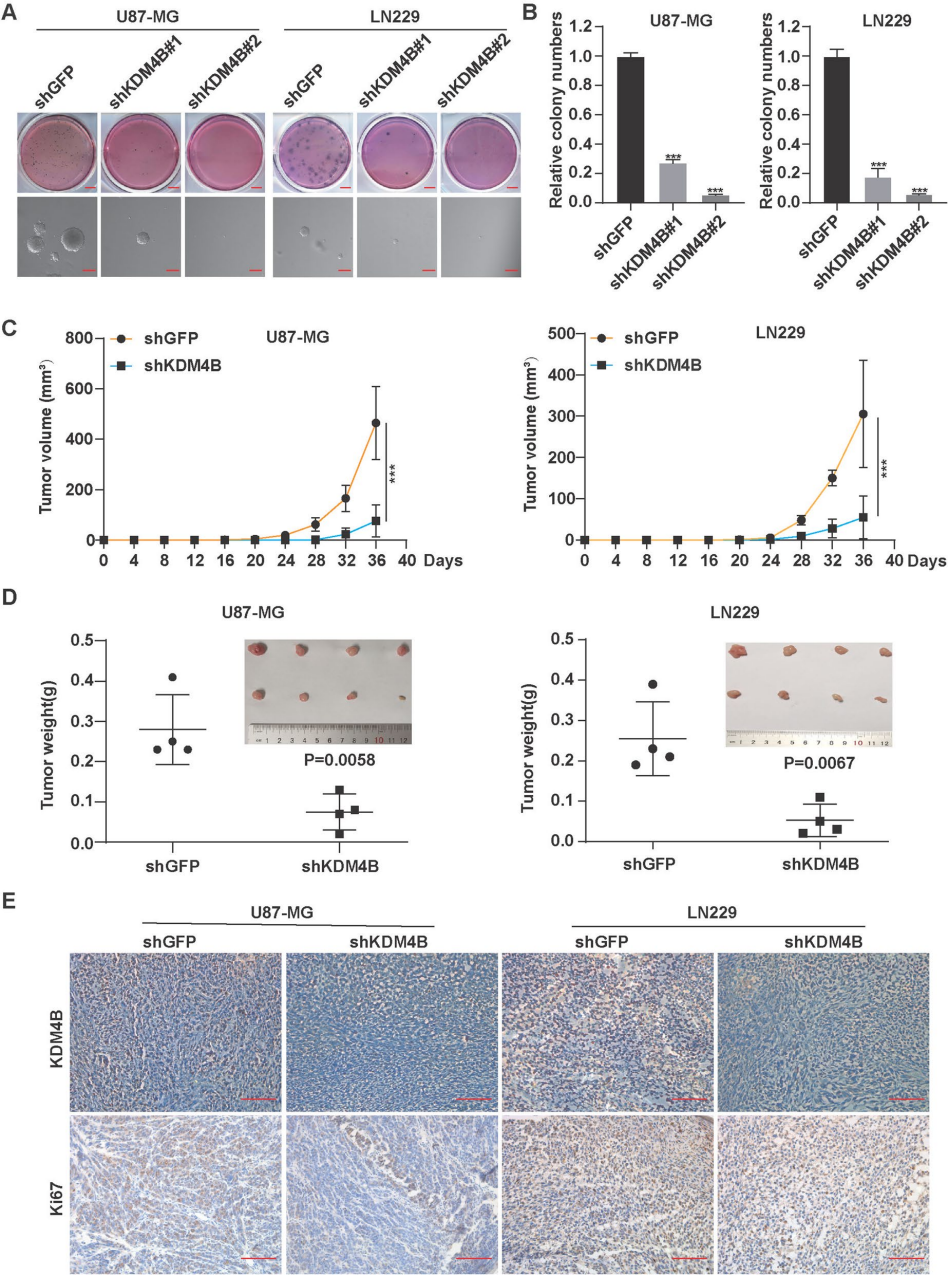
KDM4B is required for the anchorage-independent growth and tumorigenesis of GBM cells. **A, B** The colony formation ability of KDM4B-knockdown GBM cells was evaluated. **C, D** The volume and weight of the tumors were measured after KDM4B knockdown. **E** The expression levels of KDM4B and Ki67 upon KDM4B-knockdown were evaluated by immunohistochemistry in tumor tissues. ***p < 0.001

Given that abnormal expression of KDM4B is an indicator of a poor prognosis in glioma patients (Fig. 1), we investigated the effect of KDM4B on anchorage-independent cell growth and tumorigenesis. Anchorage-independent growth is one of the hallmarks of cell transformation and tumorigenesis [31], and we performed soft agar assays to examine colony formation in vitro. The results revealed that colony formation was significantly suppressed in the KDM4B knockdown group compared with the control group (Fig. 3A, B), which suggested that KDM4B knockdown markedly suppressed anchorage-independent growth. To further examine the effects of KDM4B on tumorigenicity, we generated in vivo subcutaneous xenografts in nude mice. In line with the above results, U87-MG and LN229 cells with KDM4B silencing generated much smaller tumors in immunodeficient mice than the control cells (Fig. 3C, D), indicating that KDM4B silencing suppressed the tumorigenicity of GBM cells. Consistently, KDM4B and Ki-67 expressions were also inhibited in the tumor samples (Fig. 3E). It is worth noting that the subcutaneous xenograft model has its limitations as it does not fully replicate the tumor microenvironment that GBM depends on. Nonetheless, these findings suggest that KDM4B is required for anchorage-independent cell growth and tumorigenesis in GBM.

### KDM4B silencing suppresses cancer cell survival, proliferation, migration, and invasion through the MYC signaling pathway

**Fig. 4:**
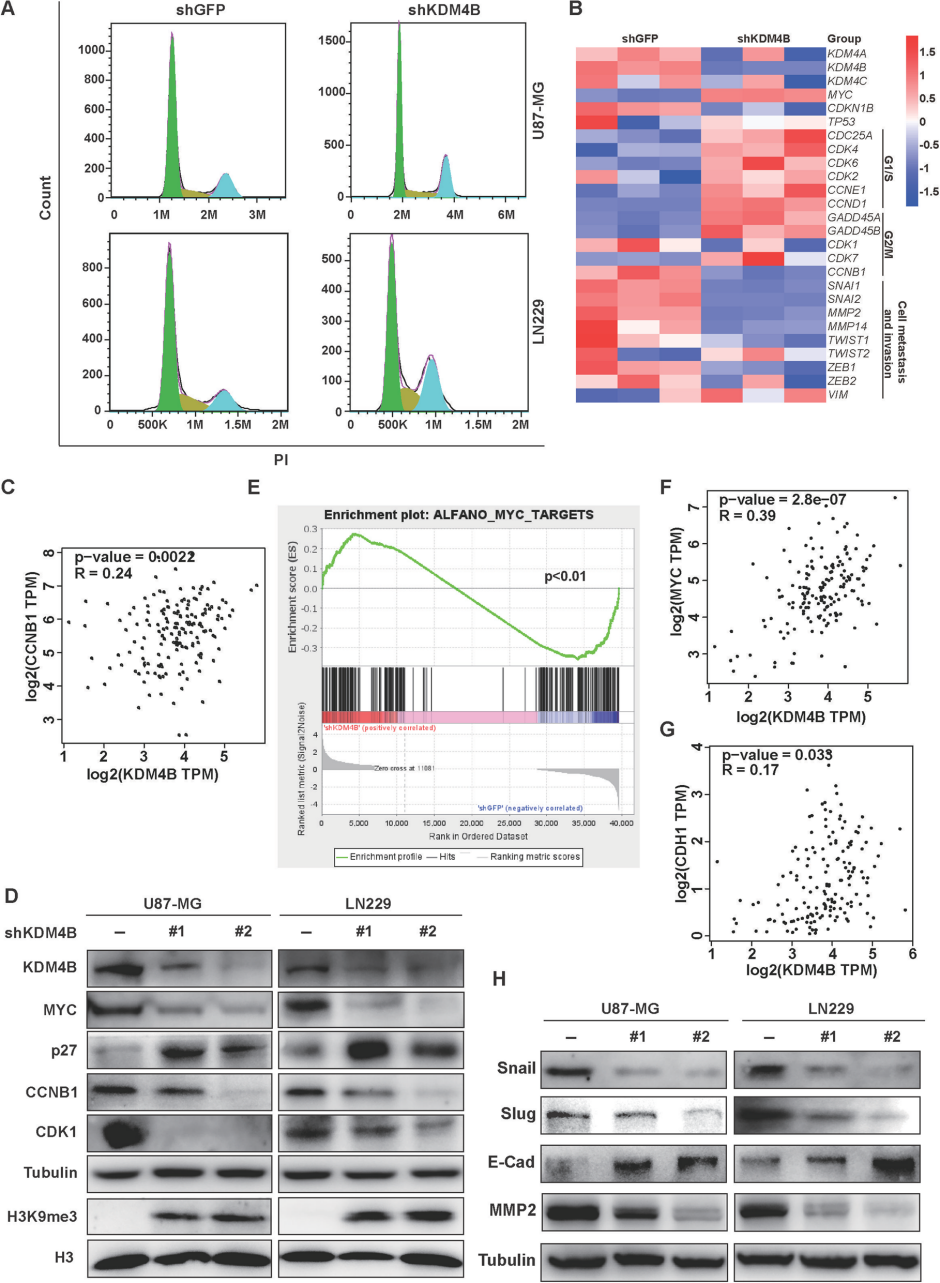
KDM4B silencing prevents cancer cell survival, proliferation, migration, and invasion through the MYC signaling pathway. **A** Flow cytometry analyses of the cell cycle in GBM cells with KDM4B knockdown. **B** Heatmap showing differential gene expression in U87-MG/shGFP or shKDM4B cells. **C** Correlation analysis of *CCNB1* with *KDM4B* was performed using the GEPIA database. **D** Western blots were applied to check the protein levels of KDM4B, MYC, p27, cyclin B1, CDK1, tubulin, H3K9me3, and H3 in KDM4B knockdown cells. **E** Transcriptome data were used to analyze MYC signaling pathway changes through GSEA V4.2.3. The gene set was downloaded from GSEA's official website (https://www.gsea-msigdb.org/gsea/msigdb/ cards/ALFANO_MYC_TARGETS). **F-G** Correlation analysis of *MYC* and *CDH1* with *KDM4B* was performed using the GEPIA database. **H** Western blots were applied to check the protein levels of Snail, Slug, E-cadherin, MMP2, and α-Tubulin in KDM4B knockdown cells

KDM4B has been found to play an important role in tumorigenesis by regulating cell cycle progression [32–34]. Here, we found by flow cytometry that GBM cells induced G2/M arrest after KDM4B knockdown (Fig. 4A and Additional file 1: Fig. S3A). To investigate the molecular basis of cell cycle arrest by KDM4B inhibition, we performed transcriptome analysis by RNA sequencing, which revealed distinctive patterns of gene expression in cells with shGFP or shKDM4B (Fig. 4B). According to the GEPIA database, the expression of the key G2/M genes *CCNB1* and *CDK1* was positively correlated with that of KDM4B (Fig. 4C and Additional file 1: Fig. S3B). Subsequently, Western blot analysis showed that the expression of CDK1 and CCNB1 was downregulated in GBM cells after KDM4B knockdown (Fig. 4D), further indicating that KDM4B might affect cell cycle progression by regulating the expression of CCNB1 and CDK1.

MYC is a major oncogene in GBM cells, and its amplification often enhances the malignant progression of cancer cells [35, 36]. Gene set enrichment analysis (GSEA) of our mRNA-seq data revealed that KDM4B silencing decreased the expression levels of most of the genes involved in the MYC signaling pathway (Fig. 4E). Moreover, the expression of MYC was positively correlated with that of KDM4B in the GEPIA database analysis (Fig. 4F). Our findings showed that KDM4B expression was positively correlated with MYC expression but negatively correlated with the expression of p27 (a downstream protein of MYC) (Fig. 4D). These data suggest that KDM4B is involved in the MYC signaling pathway. histone H3 lysine 9 trimethylation (H3K9me3) is a conserved histone modification and a repressive mark [37]. Knockdown of KDM4B in U87-MG and LN229 cells led to a significant increase in H3K9me3 levels (Fig. 4D), indicating that KDM4B regulation of gene expression is epigenetically relevant. Epithelial-to-mesenchymal transition (EMT) is crucial in tumor invasiveness and metastasis [38]. We found that the expression of the epithelial marker, *cadherin 1* (*CDH1*, encoding E-cadherin protein) was positively correlated with that of KDM4B in the GEPIA database analysis (Fig. 4G). Furthermore, the levels of the EMT-related protein Snail/Slug were significantly decreased after KDM4B knockdown, resulting in the upregulation of E-cadherin (Fig. 4H). Matrix metalloproteinase MMP2 plays a crucial role in the migration phase of EMT during neurogenesis [39], we therefore investigated the changes in the expression of MMP2. Similarly, the expression of MMP2 was downregulated after KDM4B silencing (Fig. 4H). Together, our findings suggest that KDM4B silencing suppresses cancer cell survival, proliferation, migration, and invasion through the MYC signaling pathway.

### *KDM4B mediates the ubiquitination of MYC *via* the SCF*^*FBXL3*+*CRY2*^* E3 ligase complex*

**Fig. 5:**
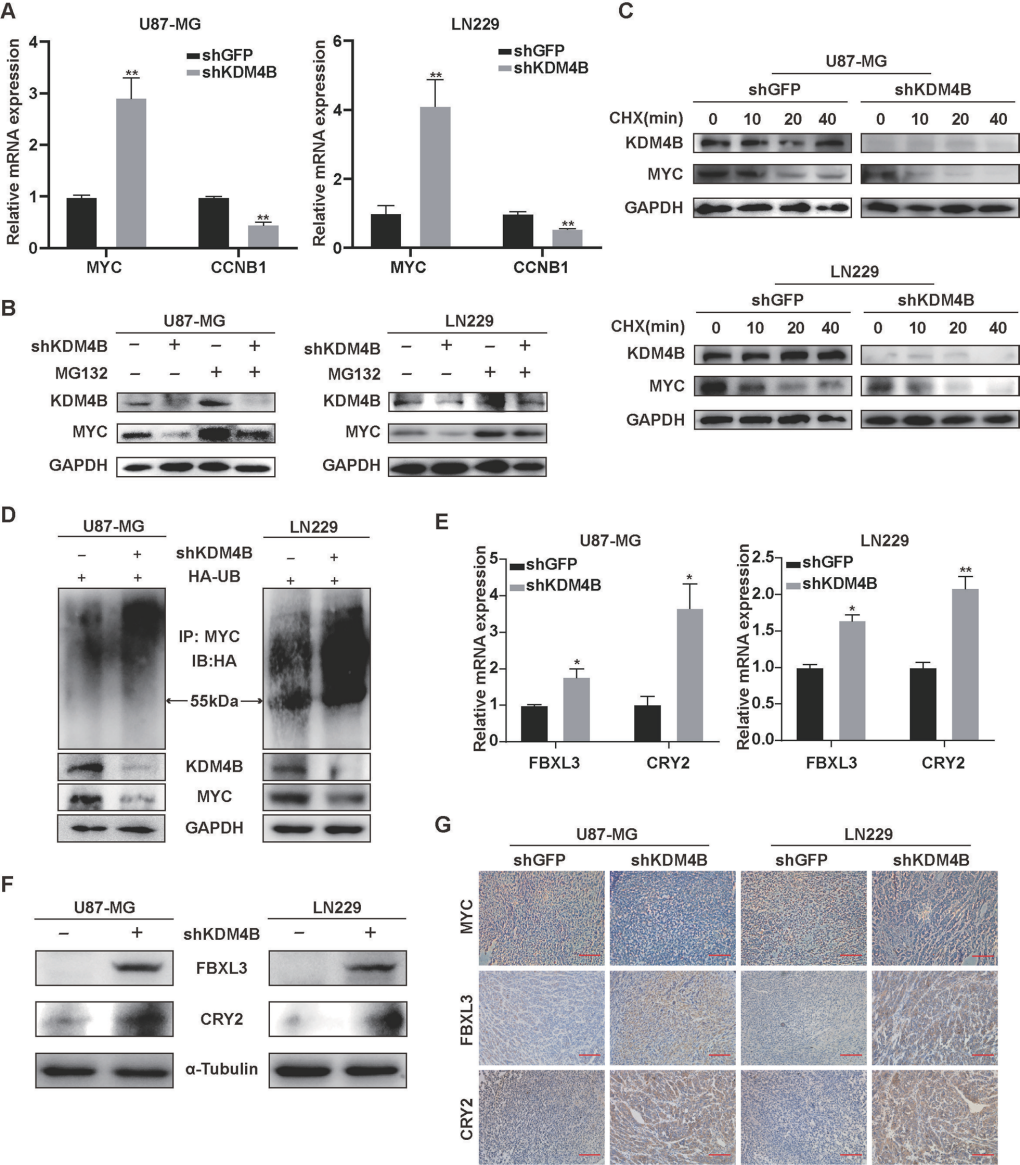
KDM4B mediates the ubiquitination of MYC via the E3 ligase complex SCF^FBXL3+CRY2^. **A** The qPCR was adopted to assess the mRNA levels of MYC and CCNB1 in KDM4B-knockdown GBM cells. **B** The shKDM4B plasmid and HA-Ub plasmid were co-transfected into U87-MG and LN229 cells, and the transfected cells were treated with MG132 for the ubiquitination assay. **C** The degradation rate of MYC was measured. U87-MG and LN229 cells were transfected with the shGFP plasmid or shKDM4B plasmid and then treated with CHX (100 μg/ml) for the indicated times. **D** Cell lysates were prepared from KDM4B-knockdown cells treated with or without MG132 for 8 h. Equal amounts of cell lysates were immunoblotted with the indicated antibodies. **E–F** The mRNA and protein levels of FBXL3 and CRY2 were measured after KDM4B knockdown in U87-MG and LN229 cells. **G** The expression levels of MYC, FBXL3, and CRY2 in response to KDM4B knockdown were evaluated by immunohistochemistry in tumor tissues. *p < 0.05, ** *p* < 0.01

The above observation indicated that KDM4B regulates the expression of the CCNB1 gene through the MYC signaling pathway. To gain further molecular understanding of the effect of KDM4B on the MYC signaling pathway, we investigated the possibility that KDM4B transcriptionally regulates the expression of CCNB1 and MYC. Quantitative PCR analysis revealed that KDM4B downregulated the mRNA expression of CCNB1 but upregulated that of MYC, suggesting that KDM4B may regulate MYC levels post-transcriptionally (Fig. 5A). We therefore explored the effect of KDM4B on the stability of the total MYC protein. KDM4B silenced cells were treated with the proteasomal inhibitor MG-132, and the MYC protein level was restored (Fig. 5B), indicating that KDM4B post-transcriptionally regulates MYC expression. Furthermore, KDM4B silencing markedly increased the rate of protein degradation in GBM cells treated with the de novo protein synthesis inhibitor cycloheximide (CHX) (Fig. 5C). To gain further understanding of KDM4B-mediated MYC stabilization, we performed in vitro ubiquitination assays. The results showed that KDM4B silencing significantly promotes the ubiquitination of MYC (Fig. 5D). To determine whether E3 ubiquitin ligases are involved in KDM4B-mediated MYC stabilization, we assessed the mRNA expression of the E3 ligase of MYC after KDM4B knockdown (data not shown). And we found that the E3 ligase complex SCF^FBXL3+CRY2^ components FBXL3 and CRY2 were significantly upregulated at the mRNA level (Fig. 5E). It has been reported that the circadian clock component CRY2 is an essential cofactor in the SCF^FBXL3^-mediated ubiquitination of MYC [40]. Similarly, the protein levels of both FBXL3 and CRY3 protein expression were markedly increased (Fig. 5F). Immunohistochemical analysis of tumor tissues showed that MYC expression was downregulated after KDM4B knockdown, while FBXL3 and CRY2 expression was upregulated (Fig. 5G). Collectively, these results indicate that KDM4B mediates the ubiquitination of MYC via the E3 ligase complex SCF^FBXL3+CRY2^.

### KDM4B interacts with MYC to promote cell proliferation for epigenetic activation

**Fig. 6:**
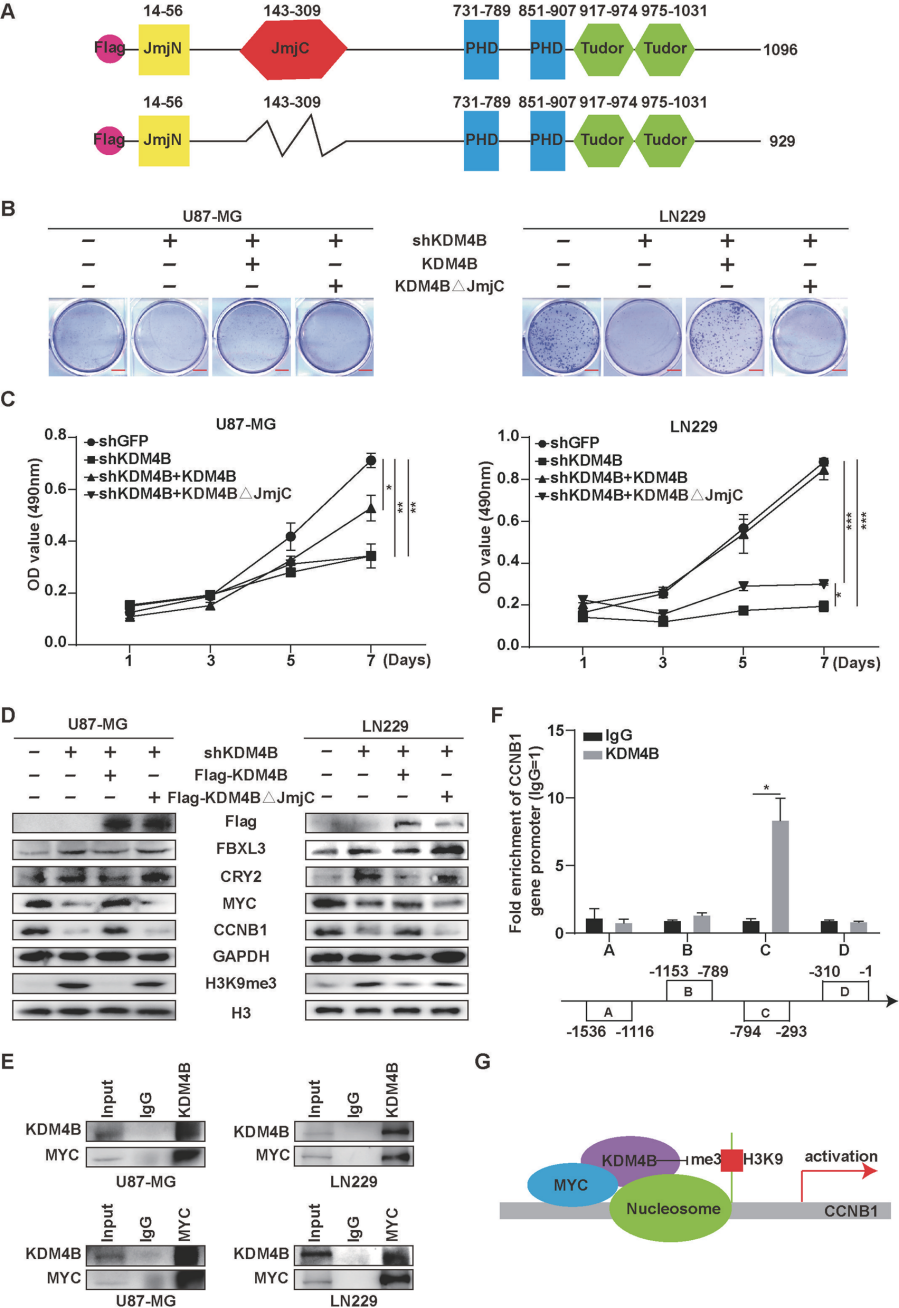
KDM4B interacts with MYC to promote cell proliferation for epigenetic activation. **A** Wild-type and mutant KDM4B plasmids with Flag tags were constructed. The KDM4B mutant contained multiple point mutations in the JmjC region. **B** Plate colony formation assays were used to evaluate the colony formation ability of GBM cells after restoration of wild-type KDM4B or mutant KDM4B expression. **C** MTT assays were used to determine the proliferative capacity of GBM cells after restoration of wild-type KDM4B or mutant KDM4B. **D** Western blots were applied to evaluate the levels of Flag, FBXL3, CRY2, MYC, cyclin B1, GAPDH, H3K9me3, and H3 after restoration of wild-type KDM4B or mutant KDM4B. **E** IP assays were used to detect the interaction between KDM4B and C-MYC in GBM cells. **F** ChIP assays were used to detect the binding of KDM4B to the promoter of CCNB1. **G** Schematic diagram of the mechanism by which KDM4B participates in the regulation of CCNB1. *p < 0.05, **p < 0.01, ***p < 0.001

**Fig. 7:**
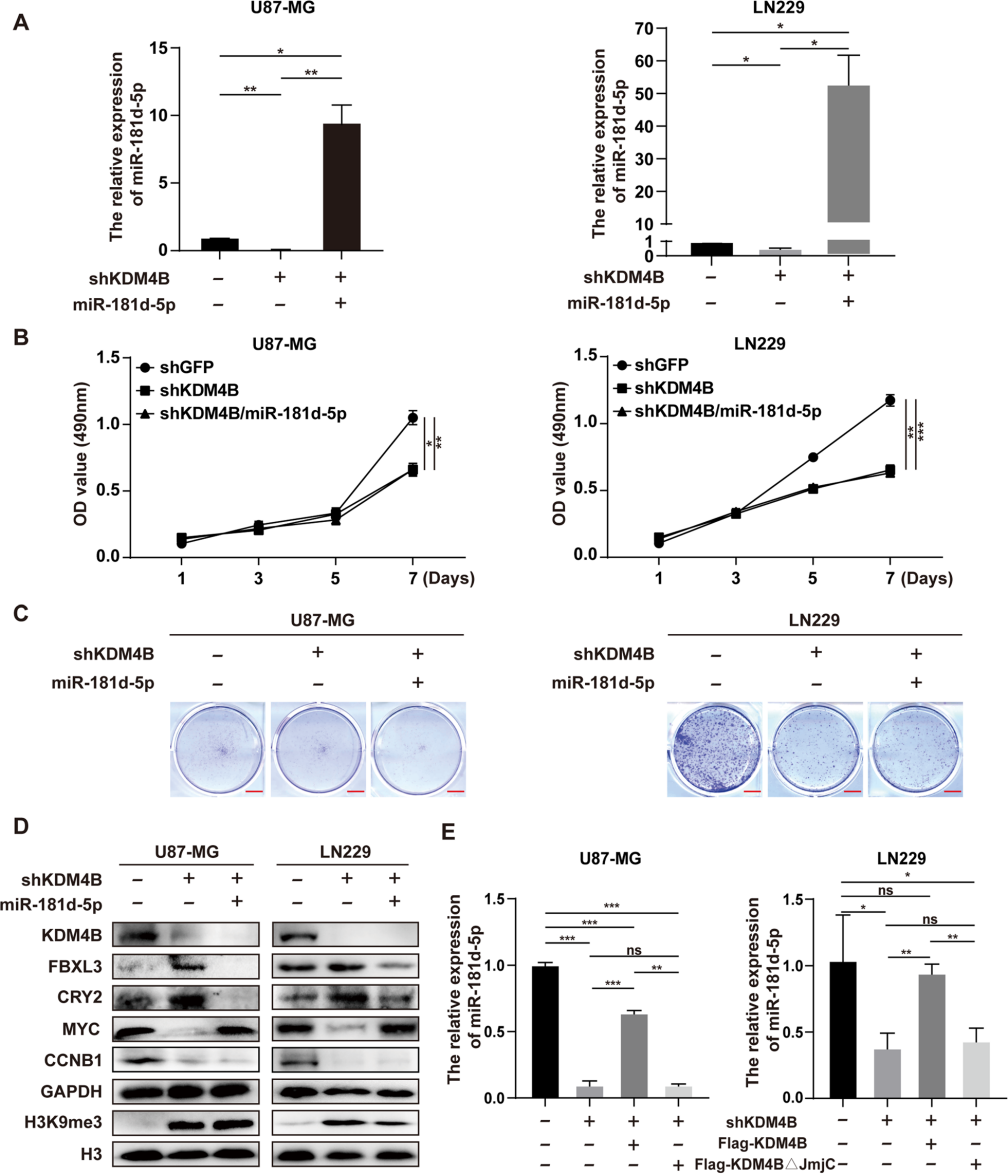
KDM4B promotes cell proliferation through the miR-181d-5p/SCF^FBXL3+CRY2^/MYC axis. **A** miR-181d-5p was detected by microRNA detection kits. **B** MTT assays were used to evaluate the proliferative capacity of GBM cells after miR-181d-5p was transfected. **C** Plate colony formation assays were used to evaluate the colony formation ability of GBM cells after miR-181d-5p was transfected. **D** Western blots were applied to measure the protein levels of KDM4B, FBXL3, CRY2, MYC, cyclin B1, GAPDH, H3K9me3, and H3 after miR-181d-5p was transfected. **E** The expression of miR-181d-5p was measured after restoration of wild-type KDM4B or mutant KDM4B. *p < 0.05, **p < 0.01, ***p < 0.001

To gain further understanding of the molecular basis of KDM4B epigenetic action, we constructed a plasmid to express KDM4B with mutation of the JmjC domain (Fig. 6A). When the expression of KDM4B was restored after KDM4B knockdown, the single-cell colony formation ability of GBM cells was restored, but this restoration did not occur if the JmjC domain was mutated (Fig. 6B and Additional file 1: Fig. S4). The MTT assay also indicated a positive correlation between GBM cell proliferation and normal KDM4B function (Fig. 6C). Further Western blot analysis showed that the basal expression of KDM4B was restored, but the expression of FBXL3 and CRY2 was downregulated by overexpression of KDM4B but not KDM4BΔJmjC. Importantly, the protein levels of MYC and CCNB1 were restored by overexpression of KDM4B but not KDM4BΔJmjC, accompanied by a marked reduction in H3K9me3 levels (Fig. 6D). Therefore, we speculated that KDM4B may play a key role in the regulation of CCNB1 by MYC. We found that MYC could be co-immunoprecipitated with KDM4B and that KDM4B could physically interact with MYC (Fig. 7E). Moreover, the ChIP assay showed that KDM4B can bind to the promoter of CCNB1. KDM4B overexpression led to a significant increase in KDM4B levels at the CCNB1 promoter (-794 ~ -293) (Fig. 6F). Taken together, these findings suggest that MYC could recruit and interact with KDM4B to *CCNB1* gene promoters for epigenetic regulation of the cell cycle (Fig. 6G).

### *KDM4B promotes cell proliferation through the miR-181d-5p/SCF*^*FBXL3*+*CRY2*^*/MYC axis*

The microRNA (miRNA) miR-181d-5p was previously found to simultaneously target FBXL3 and CRY2 mRNA to inhibit their expression and eventually up-regulate MYC [21]. Therefore, we used miRNA detection kits to assess the expression of miR-181d-5p. After KDM4B knockdown, the expression of miR-181d-5p was significantly decreased, but it was restored after overexpression of miR-181d-5p (Fig. 7A). Interestingly, the proliferation and colony formation ability of GBM cells were not restored after overexpression of miR-181d-5p (Fig. 7B, C and Additional file 1: Fig. S5). We have shown that KDM4B is required for GBM tumorigenesis, and these results demonstrated that KDM4B is the upstream regulatory gene of miR-181d-5p. Further Western blot analysis showed that in cells with KDM4B knockdown and miR-181d-5p transfection, the expression levels of FBXL3 and CRY2 were indeed decreased, but the protein expression of MYC was restored (Fig. 7D). These results indicated that KDM4B regulates MYC stability through miR-181d-5p and the E3 ligase complex SCF^FBXL3+CRY2^ in GBM cells. However, the protein level of CCNB1 did not change with that of MYC and remained low, which suggested that KDM4B is required for MYC-induced cell proliferation (Fig. 7D). To gain an understanding of miR-181d-5p regulation by KDM4B, we investigated the possibility that KDM4B epigenetically promotes the expression miR-181d-5p. After KDM4B silencing, we found that the expression of miR-181d-5p was restored by overexpression of KDM4B but not KDM4BΔJmjC (Fig. 7E). These results suggest that the ability of KDM4B to transcriptionally activate miR-181d-5p depends on its demethylase activity, which indicates that KDM4B epigenetically activates the transcription of miR-181d-5p. These findings, together with those presented above, indicate that KDM4B promotes cell proliferation through the miR-181d-5p/SCF^FBXL3+CRY2^/MYC axis.

## Discussion

GBM is one of the most prevalent primary malignant brain tumor in adults, and the prognosis for patients is exceedingly poor [1, 3, 4]. One potential approach is to identify suitable therapeutic targets in order to develop effective GBM treatment methods for GBM [4]. Abnormal expression of KDM4B, a histone demethylase, is often considered an indicator of a poor prognosis in many cancers [8]. However, there have been limited studies on KDM4B in GBM. Our findings demonstrate that KDM4B, acting as an oncogene in GBM, can suppress cell proliferation and induce G2/M arrest in GBM, and also diminish their migration and invasion abilities. Moreover, we found that KDM4B-mediated GBM cell proliferation was related to the JmjC domain. The JmjC domain is the main active domain of KDM4B and mainly catalyzes H3K9me3 demethylation, thus inducing transcriptional activation of downstream genes [41].

**Fig. 8:**
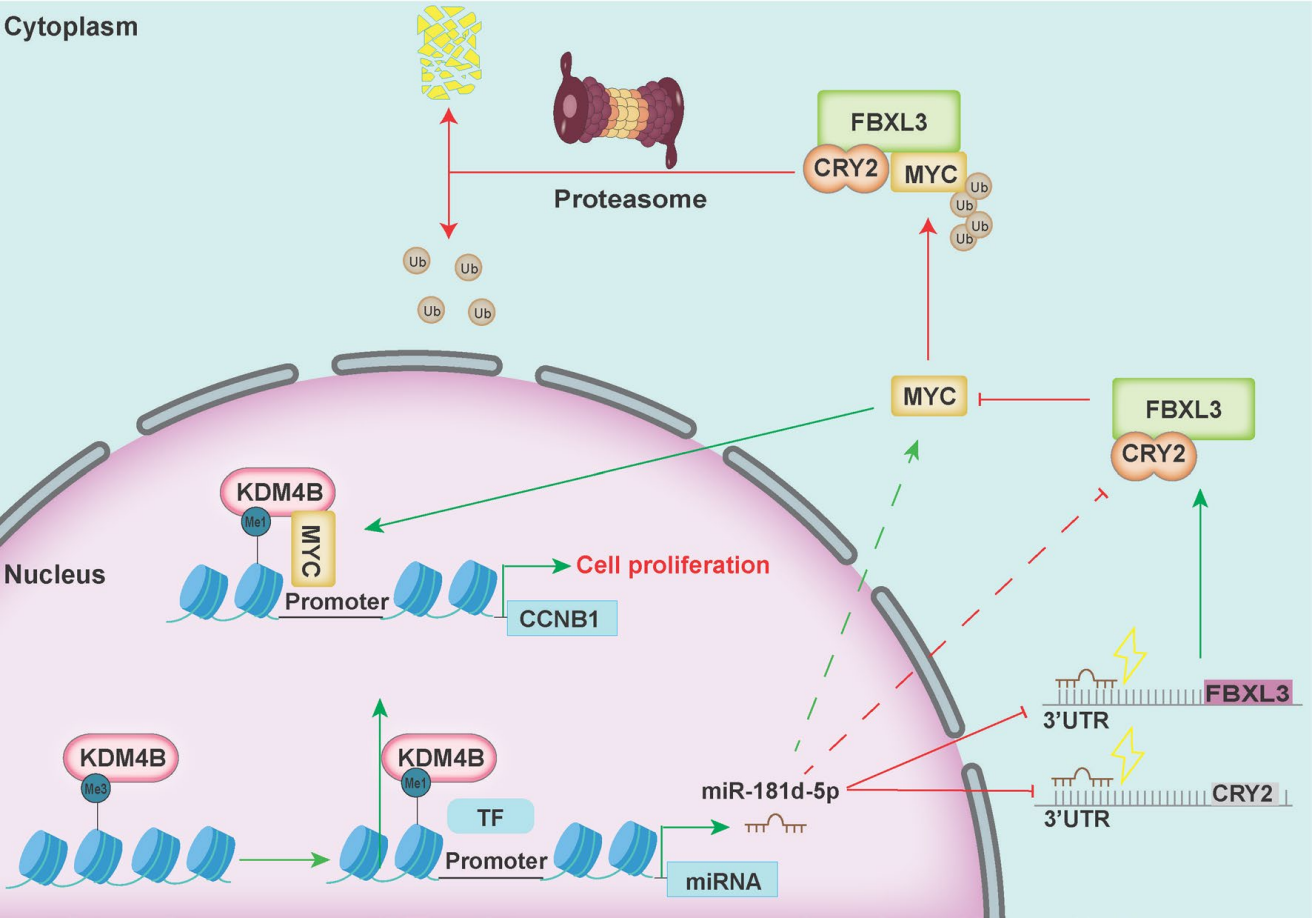
Model for KDM4B-MYC cooperation in regulating cell proliferation in GBM cells

The MYC protein is a transcription factor in the MYC gene family that mainly plays a role in transcriptional regulation and provides a platform for the binding of other cofactors, thus leading to the pleiotropic effect of MYC [16]. In GBM, MYC plays a role in promoting proliferation and metabolic reprogramming and enhancing chemotherapeutic resistance [17]. Therefore, strict restriction of MYC expression in GBM could have great therapeutic value. KDM4B overexpression and knockdown experiments showed that MYC protein expression was positively correlated with KDM4B expression. Previous studies have confirmed that KDM4B can transcriptionally activate MYC expression by recruiting by androgen receptor (AR) or estrogen receptor (ER) [10, 12, 42]. However, our results indicated that KDM4B might regulate MYC expression through posttranslational modifications in GBM. This phenomenon has not been observed in previous studies, because the expression of AR and ER is lower in GBM tissues than in reproductive system tissues. FBXL3 is a MYC ubiquitin ligase that requires the presence of CRY2 to properly ubiquitinate MYC, which is an essential cofactor in the SCF^FBXL3^-mediated ubiquitination of MYC [19, 40]. Our findings, together with those from recent studies on FBXL3 and CRY2 [19, 21, 40, 43], indicate that KDM4B regulates the ubiquitination of MYC through the E3 ligase complex SCF^FBXL3+CRY2^. A previous study has identified an epigenetic mechanism in which miR-181d-5p targets FBXL3 and CRY2, consequently stabilizing the MYC protein [21]. Consistently, our findings demonstrated that miR-181d-5p expression is positively correlated with KDM4B and MYC expression. Next, we overexpressed miR-181d-5p after KDM4B knockdown, which led to a markedly restoration of MYC expression. However, overexpression of miR-181d-5p could not restore CCNB1 expression or cell viability, indicating that KDM4B is required for miR-181d-5p action. Moreover, our data demonstrated that the ability of KDM4B to transcriptionally activate miR-181d-5p depends on its demethylase activity, which indicates that KDM4B epigenetically activates the transcription of miR-181d-5p. Collectively, our findings indicate that KDM4B promotes tumor progression through the miR-181d-5p/SCF^FBXL3+CRY2^/MYC axis (Fig. 8). Taken together, these data provide direct evidence that KDM4B epigenetically enhances MYC stability and shed new light on its oncogenic activity in tumor development.

## Conclusions

In summary, our findings provide clinical and experimental evidence that KDM4B acts as an epigenetic oncogene in GBM by stabilizing MYC expression. These findings identify a KDM4B-dependent epigenetic mechanism in the control of tumor progression, providing a rationale for targeting KDM4B as a potential treatment for GBM with high-level MYC amplification.

### Supplementary Information

**Additional file 1. Fig. S1.** CNV levels of KDM4B were detected between MGMT methylated and unmethylated statuses.**Fig. S2**. Effect of KDM4B knockdow on GBM cell growth, proliferation, migration and invasion. (**A**, **B**) The qPCR and Western blots were used to detect KDM4B expression in knockdown cells. (**C**) Relative cell number was measured by phase contrast image analysis after knocking down KDM4B. (**D**) The BrdU-positive cell percent was measured by BrdU incorporation assays after knocking down KDM4B. (**E**) Relative colony number was measured by plate clone formation assay after knocking down KDM4B. (**F**) The migration ability of KDM4B-knockdown GBM cells was measured by the wound healing test. **Fig. S3**. Effect of KDM4B knockdow on GBM cell cycle. (**A**) Percentage of indicated U87-MG and LN229 cells in different phase of Fig.4A. (**B**) Correlation analysis of CDK1 with KDM4B was performed through the GEPIA database. **Fig. S4**. Relative colony number was measured by plate clone formation assay after restoration of wild-type KDM4B or mutant KDM4B. **Fig. S5**. Relative colony number was measured by plate clone formation assay after miR-181d-5p was transfected.

## Data Availability

All data from this study were used for the publication of this article and are guaranteed for availability.

## Abbreviations

AR: Androgen receptor;
BHLH-LZ: Basic helix-loop-helix-leucine-zipper
CCNB1: Cyclin B1
CDH1: Cadherin 1
CDK1: Cyclin-dependent kinase 1
CHIP: HSC70-interacting protein
ChIP: Chromatin immunoprecipitation
CHX: Cycloheximide
CNV: Copy number variants
CRY2: Cryptochrome circadian regulator 2
DMSO: Dimethyl sulfoxide
EMT: Epithelial-to-mesenchymal transition
ER: Estrogen receptor
FBXL3: F-box and leucine-rich repeat protein 3
FBXW7: F-box and WD repeat domain containing 7
GAPDH: Glyceraldehyde-3-phosphate dehydrogenase
GBM: Glioblastoma
GSEA: Gene set enrichment analysis
H3K9me3: H3 lysine 9 trimethylation
IHC: Immunohistochemical staining
IP: Immunoprecipitation
KDM4: Histone demethylase 4
KDM4B: Histone demethylase 4B
miRNA: MicroRNA
MMP2: Matrix metallopeptidase 2
MYC: Myelocytomatosis
PI: Propidium iodide
qPCR: Quantitative PCR
