# Supplementary material for: Histone demethylase KDM4B accelerates the progression of glioblastoma via the epigenetic regulation of MYC stability
Source: Clin Epigenetics. 2023 Dec 13;15:192. doi: 10.1186/s13148-023-01608-4 (PMC10720090; doi:10.1186/s13148-023-01608-4)
Supplement: Supplementary file 1 — Additional file 1. Fig. S1. CNV levels of KDM4B were detected between MGMT methylated and unmethylated statuses.Fig. S2. Effect of KDM4B knockdow on GBM cell growth, proliferation, migration and invasion. (A, B) The qPCR and Western blots were used to detect KDM4B expression in knockdown cells. (C) Relative cell number was measured by phase contrast image analysis after knocking down KDM4B. (D) The BrdU-positive cell percent was measured by BrdU incorporation assays after knocking down KDM4B. (E) Relative colony number was measured by plate clone formation assay after knocking down KDM4B. (F) The migration ability of KDM4B-knockdown GBM cells was measured by the wound healing test. Fig. S3. Effect of KDM4B knockdow on GBM cell cycle. (A) Percentage of indicated U87-MG and LN229 cells in different phase of Fig.4A. (B) Correlation analysis of CDK1 with KDM4B was performed through the GEPIA database. Fig. S4. Relative colony number was measured by plate clone formation assay after restoration of wild-type KDM4B or mutant KDM4B. Fig. S5. Relative colony number was measured by plate clone formation assay after miR-181d-5p was transfected. [file 13148_2023_1608_MOESM1_ESM.docx]

**Supplementary materials**


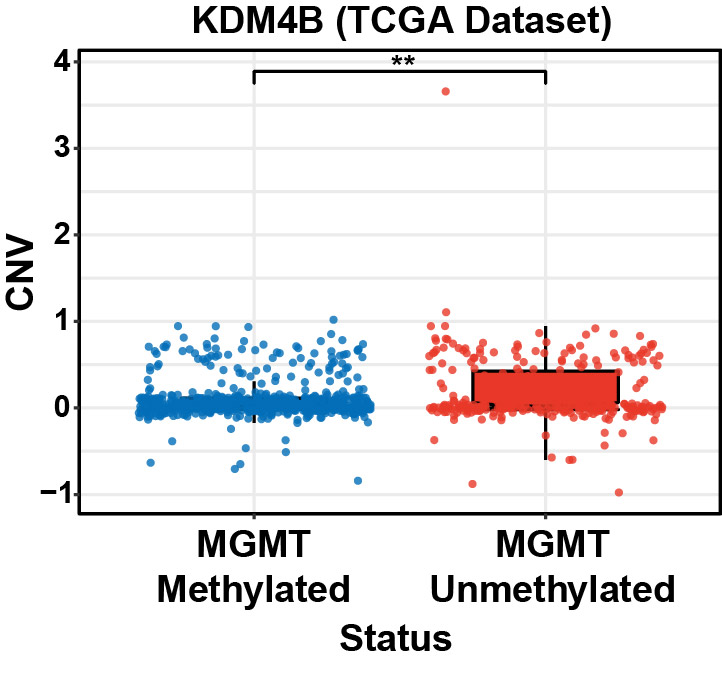


**Supplementary Fig. S1** CNV levels of KDM4B were detected between MGMT methylated and unmethylated statuses.


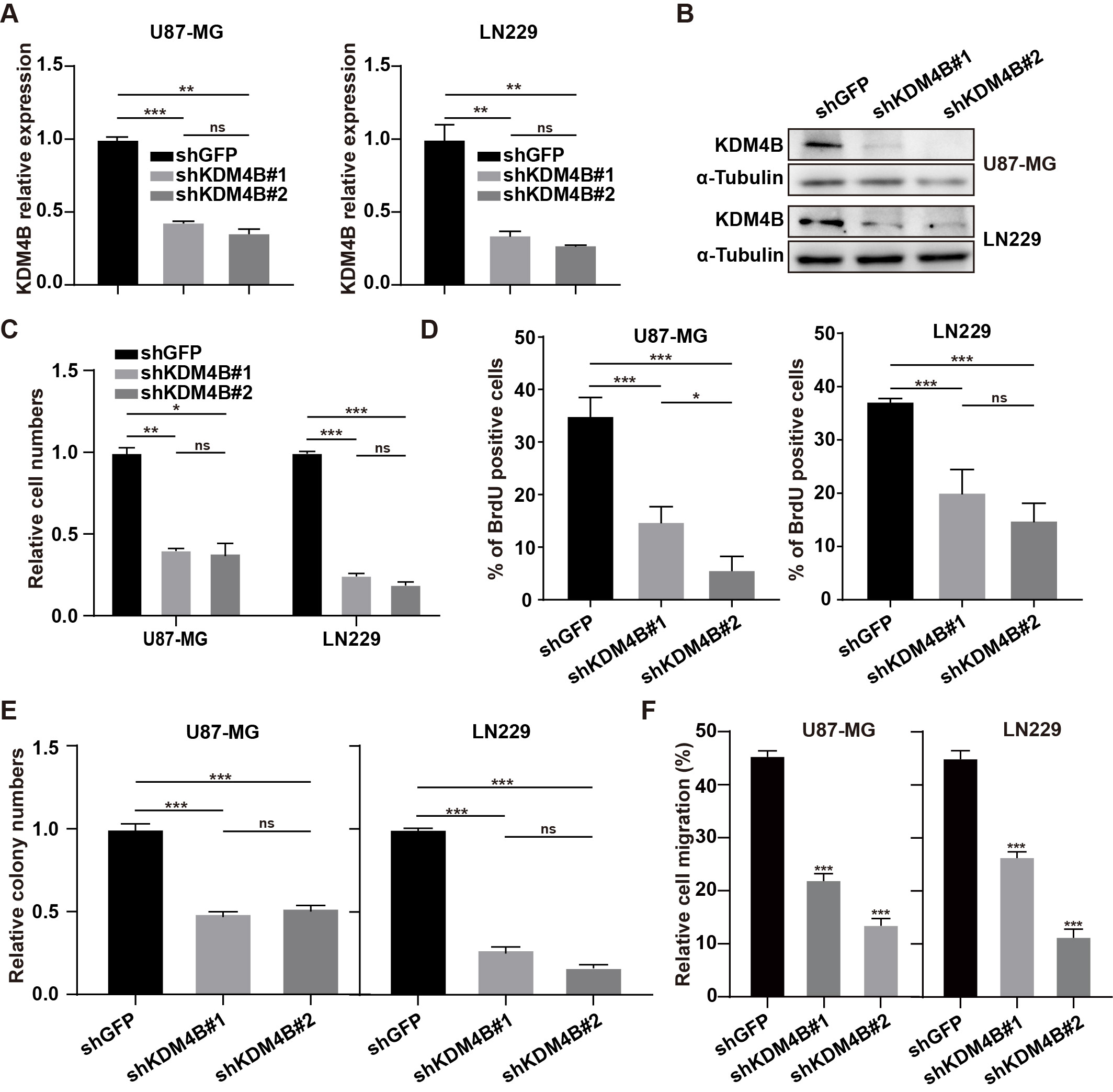


**Supplementary Fig. S2** Effect of KDM4B knockdow on GBM cell growth, proliferation, migration and invasion**.** (A-B) The qPCR and Western blots were used to detect KDM4B expression in knockdown cells. (C) Relative cell number was measured by phase contrast image analysis after knocking down KDM4B. (D) The BrdU-positive cell percent was measured by BrdU incorporation assays after knocking down KDM4B. (E) Relative colony number was measured by plate clone formation assay after knocking down KDM4B. (F) The migration ability of KDM4B-knockdown GBM cells was measured by the wound healing test.


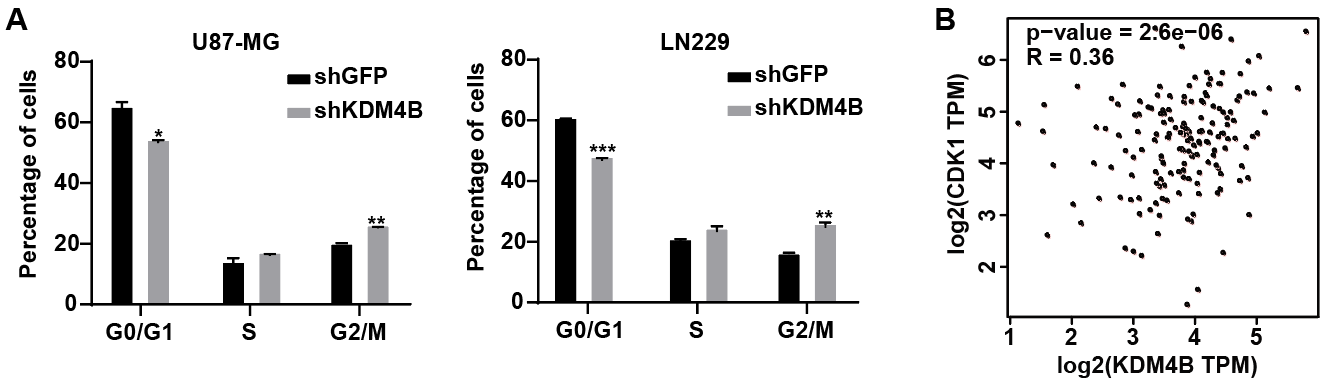


**Supplementary Fig. S3** Effect of KDM4B knockdow on GBM cell cycle. (A) Percentage of indicated U87-MG and LN229 cells in different phase of Fig.4A. (B) Correlation analysis of *CDK1* with *KDM4B* was performed through the GEPIA database.


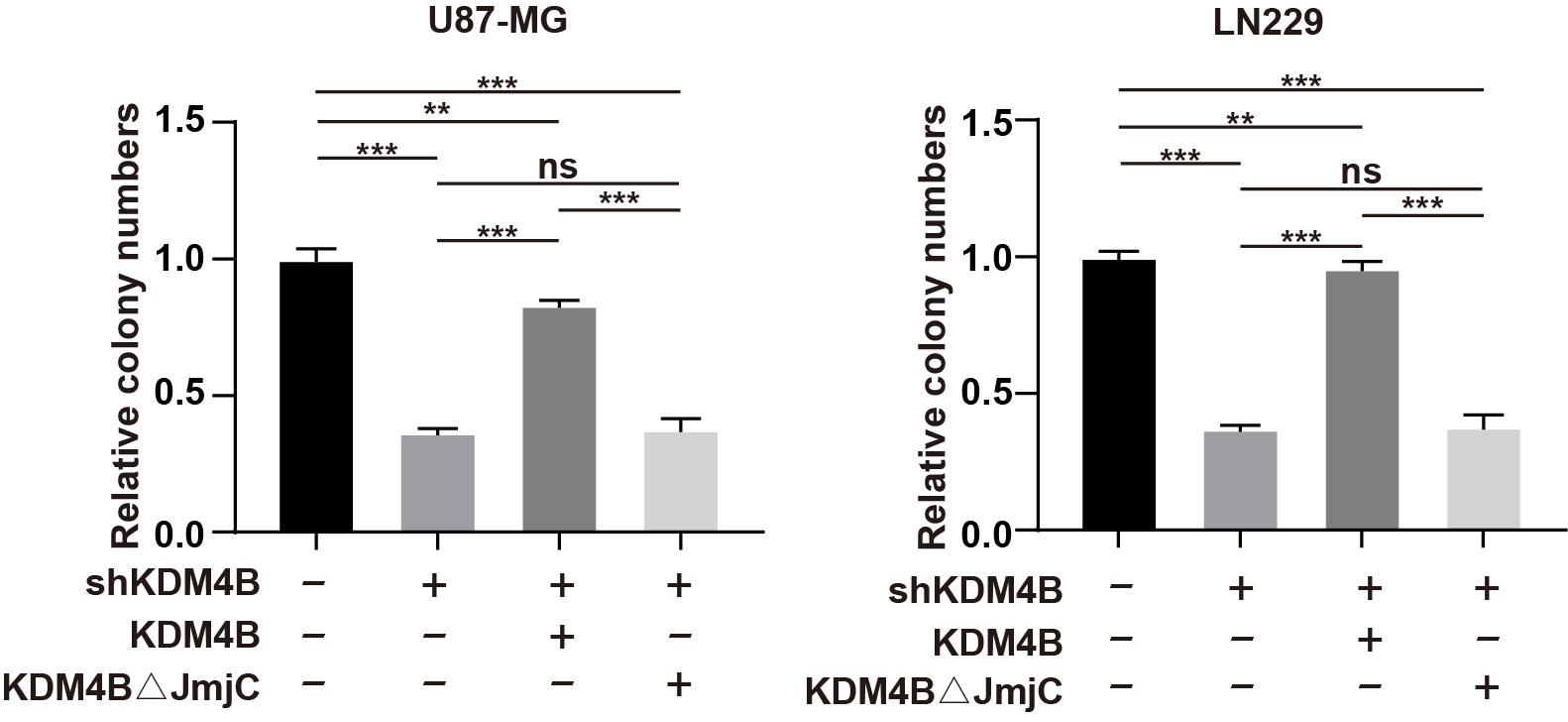


**Supplementary Fig. S****4** Relative colony number was measured by plate clone formation assay after restoration of wild-type KDM4B or mutant KDM4B.


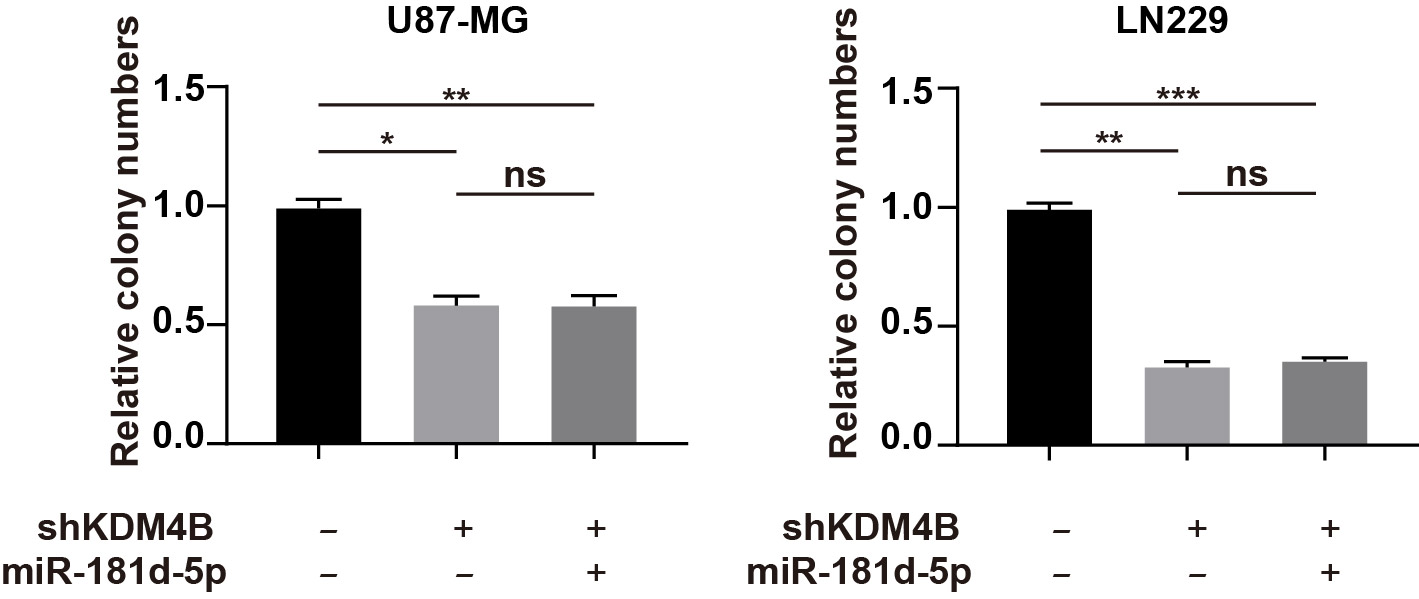


**Supplementary Fig. S5** Relative colony number was measured by plate clone formation assay after miR-181d-5p was transfected.
